# Physiological Experiments on a Person Decapitated

**Published:** 1880-02

**Authors:** 


Physiological Experiments on a Person Decapitated.—Observations made on a criminal beheaded by the guillotine, and communicated by M. Decaisne, show that both sensation and life are extinct in five minutes. The brain presented nothing of interest more than a rather bloodless condition on section. All the muscles responded to electricity for an hour and a half after execution, or up to the moment when the remains were taken in charge by the grave diggers. Various expressions could be in this way communicated to the face; the teeth could be made to grind and chatter; the eyes to roll and the eyelids to open and close; the arms to extend and flex; the hand to grasp firmly that of the experimenter; movements of artificial respiration excited, etc. The object of the experiments was to show that death is immediate on decapitation by the blade of the guillotine.—*Southern Clinic.*

